# Artificial intelligence coronary computed tomography, coronary computed tomography angiography using fractional flow reserve, and physician visual interpretation in the per-vessel prediction of abnormal invasive adenosine fractional flow reserve

**DOI:** 10.1093/ehjimp/qyae035

**Published:** 2024-05-06

**Authors:** Andrew Chiou, Melody Hermel, Rajbir Sidhu, Eric Hu, Alexander van Rosendael, Samantha Bagsic, Emem Udoh, Ricardo Kosturakis, Mohammad Aziz, Christina Rodriguez Ruiz, Shawn Newlander, Bahram Khadivi, Jason Parker Brown, Martin L Charlat, Paul S Teirstein, Curtiss T Stinis, Richard Schatz, Matthew J Price, Jeffrey Cavendish, Michael Salerno, Austin Robinson, Sanjeev Bhavnani, Jorge Gonzalez, George E Wesbey

**Affiliations:** Department of Cardiology, Scripps Clinic, 9898 Genesee Avenue, AMP 400, La Jolla, CA 92037, USA; Department of Cardiology, United Medical Doctors, La Jolla, CA, USA; Department of Cardiology, Sutter East Bay Medical Group, Oakland, CA, USA; Department of Research & Development, Biostatistics, Scripps Health, La Jolla, CA, USA; Department of Cardiology, Leiden University Medical Center, Leiden, The Netherlands; Department of Research & Development, Biostatistics, Scripps Health, La Jolla, CA, USA; Department of Internal Medicine, Bakersfield Memorial Hospital, Bakersfield, CA, USA; Department of Cardiology, El Paso Cardiology Associates, El Paso, TX, USA; Department of Cardiology, Mount Sinai/Morningside/BronxCare, Bronx, NY, USA; Department of Cardiology, MemorialCare Long Beach Medical Center, Long Beach, CA, USA; Department of Medical Physics, Scripps Health, La Jolla, CA, USA; Department of Cardiology, Scripps Prebys Cardiovascular Institute, La Jolla, CA, USA; Department of Cardiology, Scripps Prebys Cardiovascular Institute, La Jolla, CA, USA; Department of Cardiology, Scripps Prebys Cardiovascular Institute, La Jolla, CA, USA; Department of Cardiology, Scripps Clinic, 9898 Genesee Avenue, AMP 400, La Jolla, CA 92037, USA; Department of Cardiology, Scripps Clinic, 9898 Genesee Avenue, AMP 400, La Jolla, CA 92037, USA; Department of Cardiology, Scripps Clinic, 9898 Genesee Avenue, AMP 400, La Jolla, CA 92037, USA; Department of Cardiology, Scripps Clinic, 9898 Genesee Avenue, AMP 400, La Jolla, CA 92037, USA; Department of Cardiology, Scripps Prebys Cardiovascular Institute, La Jolla, CA, USA; Department of Cardiology, Stanford University, Palo Alto, CA, USA; Department of Cardiology, Scripps Clinic, 9898 Genesee Avenue, AMP 400, La Jolla, CA 92037, USA; Department of Cardiology, Scripps Clinic, 9898 Genesee Avenue, AMP 400, La Jolla, CA 92037, USA; Department of Cardiology, Scripps Clinic, 9898 Genesee Avenue, AMP 400, La Jolla, CA 92037, USA; Department of Cardiology, Scripps Clinic, 9898 Genesee Avenue, AMP 400, La Jolla, CA 92037, USA; Department of Radiology, Scripps Clinic, La Jolla, CA, USA

**Keywords:** artificial intelligence, coronary artery disease, CT-fractional flow reserve, quantitative CT coronary angiography, comparative studies

## Abstract

**Aims:**

A comparison of diagnostic performance comparing AI-QCT_ISCHEMIA_, coronary computed tomography angiography using fractional flow reserve (CT-FFR), and physician visual interpretation on the prediction of invasive adenosine FFR have not been evaluated. Furthermore, the coronary plaque characteristics impacting these tests have not been assessed.

**Methods and results:**

In a single centre, 43-month retrospective review of 442 patients referred for coronary computed tomography angiography and CT-FFR, 44 patients with CT-FFR had 54 vessels assessed using intracoronary adenosine FFR within 60 days. A comparison of the diagnostic performance among these three techniques for the prediction of FFR ≤ 0.80 was reported. The mean age of the study population was 65 years, 76.9% were male, and the median coronary artery calcium was 654. When analysing the per-vessel ischaemia prediction, AI-QCT_ISCHEMIA_ had greater specificity, positive predictive value (PPV), diagnostic accuracy, and area under the curve (AUC) vs. CT-FFR and physician visual interpretation CAD-RADS. The AUC for AI-QCT_ISCHEMIA_ was 0.91 vs. 0.76 for CT-FFR and 0.62 for CAD-RADS ≥ 3. Plaque characteristics that were different in false positive vs. true positive cases for AI-QCT_ISCHEMIA_ were max stenosis diameter % (54% vs. 67%, *P < 0.01*); for CT-FFR were maximum stenosis diameter % (40% vs. 65%, *P* < 0.001), total non-calcified plaque (9% vs. 13%, *P* < 0.01); and for physician visual interpretation CAD-RADS ≥ 3 were total non-calcified plaque (8% vs. 12%, *P* < 0.01), lumen volume (681 vs. 510 mm^3^, *P* = 0.02), maximum stenosis diameter % (40% vs. 62%, *P* < 0.001), total plaque (19% vs. 33%, *P* = 0.002), and total calcified plaque (11% vs. 22%, *P* = 0.003).

**Conclusion:**

Regarding per-vessel prediction of FFR ≤ 0.8, AI-QCT_ISCHEMIA_ revealed greater specificity, PPV, accuracy, and AUC vs. CT-FFR and physician visual interpretation CAD-RADS ≥ 3.

## Graphical Abstract

**Graphical Abstract:**
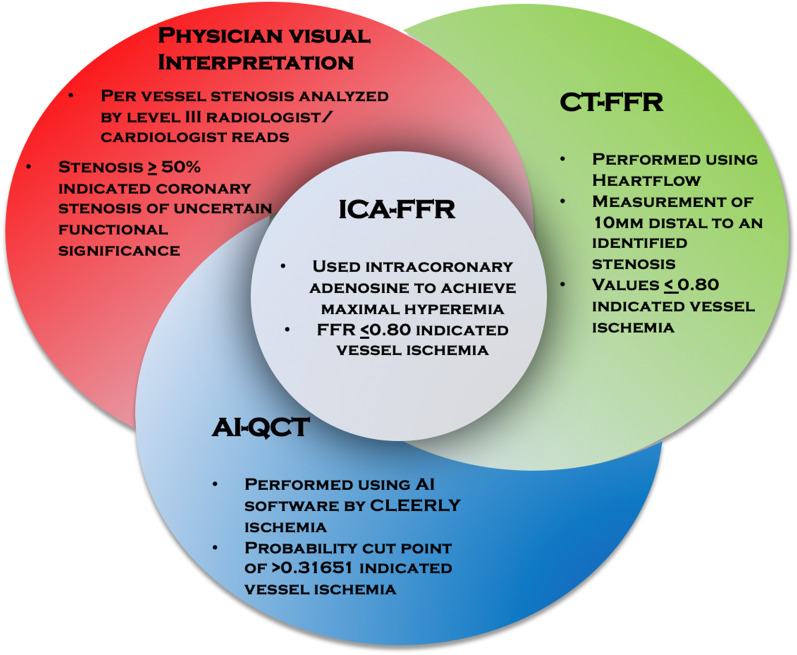

## Introduction

Coronary computed tomography angiography (CCTA) allows for direct visualization of coronary anatomy, however it is unable to estimate haemodynamic significance of stenosis with poor correlation to downstream myocardial ischaemia.

To overcome this limitation, coronary computed tomography angiography using fractional flow reserve (CT-FFR) has been developed using principles of computational fluid dynamics to simulate invasive FFR.^1^ This technique estimates the functional significance of coronary artery stenosis.^2^ Multicentre trials using CT-FFR showed greater specificity in diagnosing lesions causing ischaemia in comparison to CCTA alone.^3–6^ Still, the accuracy of CT-FFR has been variable in prior studies.^7–11^

A novel approach to further improve the diagnostic accuracy of CCTA involves machine learning. Artificial intelligence coronary computed tomography (AI-QCT_ISCHEMIA_, CLEERLY Labs, CLEERLY Inc.) is a software service that uses an artificial intelligence (AI)-based approach to interpret CCTA atherosclerosis, stenosis, and vascular morphology variables, using a series of validated convolutional neural network models. AI-QCT_ISCHEMIA_ utilizes a whole-heart 3D volumetric assessment of vascular morphology and a quantitative evaluation of atherosclerotic plaque burden and type to predict the presence of ischaemia. AI-QCT_ISCHEMIA_ outperforms stress SPECT myocardial perfusion imaging in detecting obstructive coronary artery disease (CAD) with invasive angiography as the reference standard.^12^

In a real world population with a high degree of coronary artery calcium (CAC), we performed a single centre, retrospective analysis of diagnostic performance to detect myocardial ischaemia comparing AI-QCT_ISCHEMIA_ vs. CT-FFR vs. original CCTA visual interpretation by expert physicians against gold standard adenosine invasive coronary angiography (ICA)-FFR.

## Methods

### Study protocol/population

**Figure 1:**
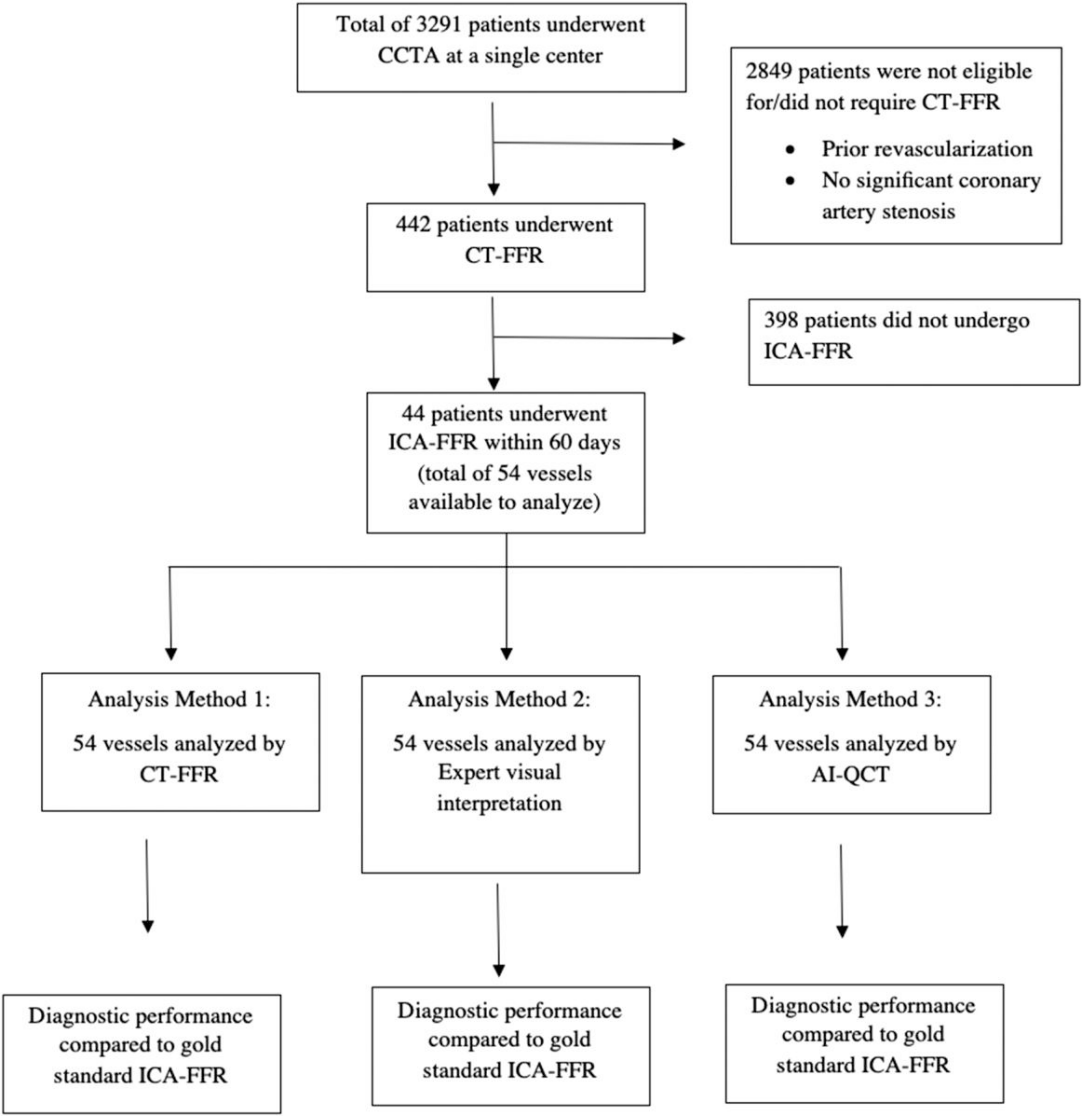
Study protocol/design.

We performed a single centre, single scanner, 43-month retrospective review of patients who underwent CCTA from 2017 to 2020. Of the patients who underwent CCTA, a subgroup of patients underwent CT-FFR based on physician visual interpretation CAD-RADS ≥ 3 and the availability of reimbursement for the CT-FFR procedure. Out of the individuals who underwent CT-FFR, we analysed a subset of patients who had ICA adenosine FFR performed within 60 days of CT-FFR based on physician preferences. At least one major vessel [right coronary artery (RCA), left anterior descending (LAD), left circumflex (LCx), and a large ramus intermedius (RI)] were measured by ICA-FFR with values between 0.5 and 0.99. Using ICA-FFR measurement as the gold standard, we compared three methods to analyse stenosis significance: expert radiologist or cardiologist visual interpretation of the CCTA, CT-FFR, and AI-QCT_ISCHEMIA_, as shown in *Figure 1*. All three methods were blinded to each other’s results. A comparison of the diagnostic performance of each of these three techniques to ICA-FFR ≤ 0.80 was reported and according to standard measurements in each technique. The Institutional Review Board approved the study protocol (IRB-17-6990). The institution paid a fee to Heartflow and CLEERLY for all analyses of CT-FFR and AI-QCT_ISCHEMIA_, respectively.

### CT imaging (CCTA, CT-FFR)

Coronary CTA was performed using a volumetric single heart beat 256 slice scanner (Revolution, GE Healthcare). Prospective gated scanning was used with a tube voltage of 100–140 kV at a current of 122–750 mA (depending on body habitus and weight) with rotation time of 0.28 s and a 16–20 cm field of view. Images were reconstructed with a slice thickness of 0.625 mm with an inter-slice gap of 0.625 mm. Studies were performed in accordance with guidelines from the Society of Cardiovascular Computed Tomography. All subjects received 0.8 mg nitroglycerine powder. Patients received i.v. or oral metoprolol for heart rate > 60 prior to undergoing CT imaging. A total of 80–90 mL Omnipaque 350 contrast was power injected at a rate of 7–8 mL/s.

The coronary calcium score (CAC) was obtained based on the total and per-vessel Agatston scores in the vessel. These scores were then correlated with both CT-FFR and adenosine FFR measurements to assess the impact of CAC on per-vessel diagnostic performance. Prospective gated scanning was used with a fixed tube voltage of 120 kV with rotation time of 0.28 s and a 25 cm field of view. Images were reconstructed with a slice thickness of 2.5 mm with an inter-slice gap of 2.5 mm. Agatston coronary calcium score per coronary territory and total coronary tree were computed by a 3D lab using Vital Images (Vitrea) software.

## Analysis method 1: CT-FFR

CT-FFR for this study was performed using Heartflow (Redwood City, CA, USA), a commercially available algorithm. CT-FFR measurement of 10 mm distal to the most severe stenosis was provided by Heartflow, and values ≤ 0.80 were deemed positive.^13^ As a sub-analysis, we compared at various thresholds including CT-FFR ≤ 0.7 and 0.7–0.8. The time necessary for analysis of CT-FFR was 6–12 h of turnaround time.

### Analysis method 2: expert physician visual interpretation of CAD-RADS

All CCTAs were contemporaneously analysed by expert radiologist or cardiologist to determine maximal stenosis severity defined by the CAD-RADS classification system.^14^ For heart rates < 70, the percent diastolic phase with the highest quality was selected and further enhanced by Snap Shot Freeze motion correction algorithm. For heart rates > 70, the percent systolic phase with the highest quality was selected and further enhanced by Snap Shot Freeze motion correction algorithm. Greater than 50% stenosis by visual assessment by expert reader was considered significant.

### Analysis method 3: AI-QCT_ISCHEMIA_

The AI-based coronary analysis of coronary CTA used in this study was performed with an AI software service (CLEERLY Labs; CLEERLY, Inc.). This software utilizes a series of validated convolutional neural network models to measure plaque characteristics, stenosis, and vessel ischaemia features. Using parameters generated by the AI-QCT_ISCHEMIA_ analysis, a novel model predicting ischaemia was developed to predict invasive FFR ≤ 0.8 on a per-vessel basis. First, vessels with AI-QCT_ISCHEMIA_-determined stenosis ≤ 20% were automatically considered non-ischaemic, and vessels with AI-QCT_ISCHEMIA_-determined stenosis > 80% were automatically considered ischaemic. For the remaining vessels, a random forest machine learning model was developed using 37 parameters from the AI-QCT_ISCHEMIA_ algorithm.^15^ The CLEERLY ISCHEMIA algorithm outputs a CLEERLY ISCHEMIA Index (CII), a binary indication of likely ischaemia regarding presence vs. absence for a given vessel, which is equivalent to invasive FFR ≤ 0.80 vs. >0.80, respectively. The probability cutpoint for ischaemia that was used was >0.31651, as previously validated.^16^ This cutpoint threshold of 0.31651 had been shown to maximize the sum of sensitivity and specificity, with the constraint that vessel-territory-level specificity had to be above 0.80. Notably, the CLEERLY ISCHEMIA algorithm is ‘locked,’ meaning it is not a continuous learning algorithm. The computational time for artificial intelligence coronary computed tomography (AI-QCT) analysis was around 11 min per case. The total time for the entire process that included quality assurance review in research was around 90 min per case.

### Gold standard: ICA-FFR

ICA was performed based on clinical indications and imaging standards. Standard coronary angiogram views were obtained to evaluate all major coronary vessels. ICA-FFR was performed on major vessels (e.g. RCA, LAD, LCx, and RI) that appeared stenotic based on visual interpretation by the interventional cardiologist. Vessels were interrogated by FFR using intracoronary (150 µg) adenosine infusion to achieve maximal hyperaemia. The primary endpoint was adenosine FFR ≤ 0.80. The interventional cardiologist was not blinded to either the physician visual CCTA interpretation or CT-FFR. The AI-QCT_ISCHEMIA_ study was performed after the cardiac catheterization.

### LAD sub-analysis

Given its critical clinical significance, we performed a sub-analysis that specifically assessed the LAD including the proximal and mid segments. We compared the diagnostic accuracy, sensitivity, and specificity among the various modalities focusing on the LAD segment.

### Assessment of plaque characteristics

Plaque characteristics were interpreted from CCTA images on per-FFR-vessel basis for the following: low density (<30 HU) plaque (%), lumen volume, maximum stenosis diameter (%), total calcified (>350 HU) plaque (%), total non-calcified (30–350 HU) plaque (%), and total plaque (%). These were obtained using the CLEERLY plaque analysis software. Plaque percentage burden was calculated as follows for each FFR vessel: vessel plaque volume (mm^3^)/vessel volume (mm^3^) × 100%.

### Statistical analysis

Analysis was performed using R v. 4.0.3 in the R Studio software environment using the following packages: e1071, caret, epiR, pROC, and car. The diagnostic accuracy, sensitivity, specificity, positive predictive value (PPV), negative predictive value (NPV), and ROC were calculated using invasive FFR as the reference standard on a per-vessel basis. The values were analysed as categorical values. The *P* value for ROC curves were calculated using the DeLong method. As AI-QCT_ISCHEMIA_ used a binary cut-off vs. the binary invasive FFR result, the McNemar test and null information rate (NIR) were conducted as part of the analyses. The NIR and McNemar test were used to determine the significance of the similarity and the difference in comparison to the gold standard (ICA-FFR), respectively. Cut-off for statistical significance was a *P* value of 0.05. A Pearson correlation analysis and Bland–Altman analysis was conducted when comparing CT-FFR against ICA-FFR, which will be provided in the Appendix, however, will not be discussed further in this manuscript as we are unable to use this test to compare AI-QCT and visual interpretation CAD-RADS against ICA-FFR.

## Results

### Study population

Out of a total of 3291 CCTA patients, a subset of 442 patients were sent for CT-FFR analysis from 2017 to 2020. These 442 patients sent for CT-FFR analysis were accepted with no rejections for image quality (442/442 patients accepted, all vessels, 100%). Of these 442 patients, 44 individuals had ICA adenosine FFR performed within 60 days in one of the major coronary arteries. The primary endpoint of adenosine FFR ≤ 0.80 was found in 19 of 54 vessels (35%).

### Baseline characteristics

Baseline characteristics of the 44 patients who underwent ICA-FFR are shown in *Table 1*. Of the study population, 76.9% were male, 24.3% had diabetes mellitus, 66.7% had hypertension, 87.2% had hyperlipidaemia, and 24.4% had current tobacco use. The mean age of the study population was 65.8 [standard deviation (SD) 8.0]. Median and mean heart rate during CT imaging were both 60 beats per minute [quartile (Q)1, Q3: 55, 65; SD 6.7].

**Table 1:** Baseline characteristics

Baseline characteristic	Percentage of study population (*n* = 44)
Male	76.9%
Diabetes mellitus	24.3%
Hypertension	66.7%
Hyperlipidaemia	87.2%
Tobacco use	24.4%

### CAC analysis

**Figure 2:**
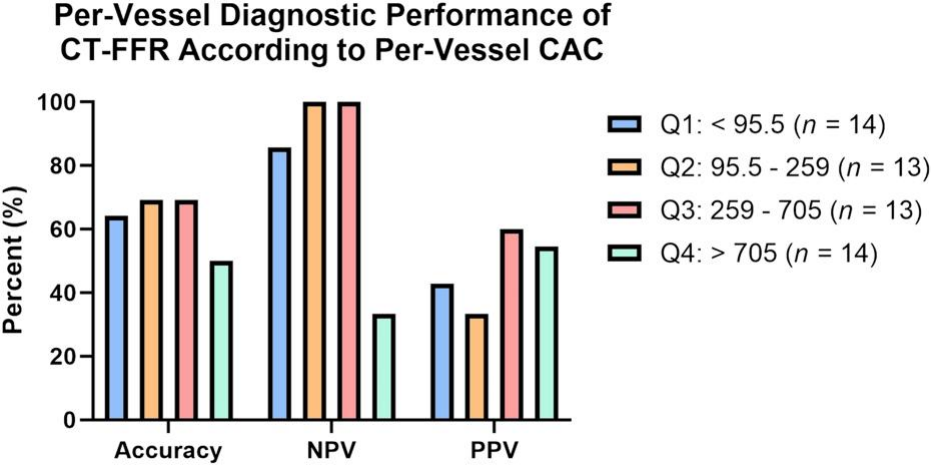
Per-vessel diagnostic performance of CT-FFR based on per-vessel CAC. CT-FFR, coronary computed tomography angiography using fractional flow reserve; CAC, coronary artery calcium; NPV, negative predictive value; PPV, positive predictive value; Q, quartile.

The mean total CAC was 1006 (SD 1021) with a median 654 (Q1, Q3: 218, 1142). Median per-vessel CAC was 259 (Q1, Q3: 95.5, 705) for the 54 vessels that underwent both CT-FFR and adenosine FFR. Nineteen subjects had a total Agatston score over 1000. Thirty-three vessels exhibited lesion-specific positive CT-FFR, of which 16 were true positive at invasive FFR. Twenty-one vessels exhibited lesion-specific negative CT-FFR, of which 18 were true negative at invasive FFR. The per-vessel diagnostic performance of CT-FFR based on per-vessel CAC is shown in *Figure 2*.

### Diagnostic performance

AI-QCT_ISCHEMIA_ revealed a higher per-vessel diagnostic accuracy, specificity, and PPV than CT-FFR and physician visual interpretation. AI-QCT_ISCHEMIA_ and CT-FFR had comparable sensitivity. These values can be found in *Table 2*.

**Table 2:** Comparing per-vessel imaging modalities vs. adenosine ICA-FFR

Metric (95% confidence interval)	Physician visual interpretation CAD-RADS ≥ 3	CT-FFR	AI-QCT_ISCHEMIA_
Diagnostic accuracy	0.46 (0.33–0.60)	0.63 (0.49–0.76)	0.78 (0.66–0.89)
Sensitivity TP/(TP + FN)	1.00 (1.00) 19/19	0.84 (0.60–0.97) 16/19	0.84 (0.65–0.97) 16/19
Specificity TN/(FP + TN)	0.17 (0.07–0.36) 6/35	0.51 (0.34–0.69) 18/35	0.74 (0.57–0.88) 26/35
Positive predictive value (PPV) TP/(TP + FP)	0.40 (0.36–0.43) 19/48	0.48 (0.39–0.58) 16/33	0.64 (0.54–0.79) 16/25
Negative predictive value (NPV) TN/(TN + FN)	1.00 (1.00) 6/6	0.86 (0.70–0.95) 18/21	0.90 (0.75–0.96) 26/29
Area under the curve (AUC)	0.62 (0.48–0.75)	0.76 (0.62–0.90)	0.91 (0.82–1.00)

TP, true positive; TN, true negative; FP, false positive; FN, false negative.

**Figure 3:**
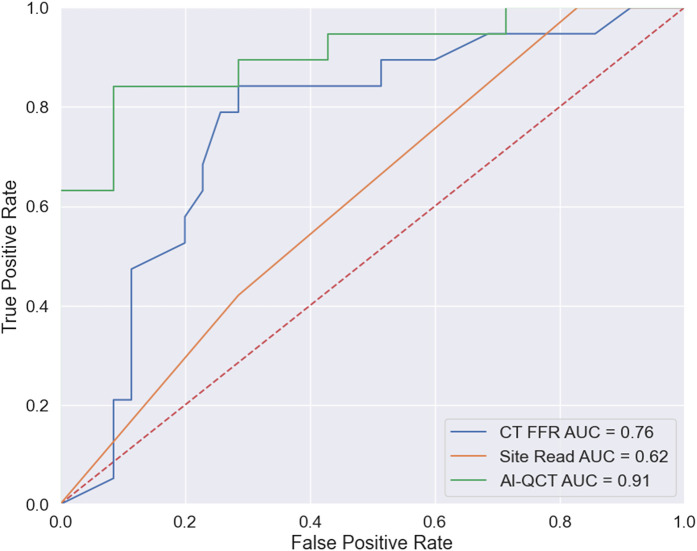
ROC curves displaying diagnostic performance of three modalities against the gold standard ICA. *P* values for AUC: CT-FFR vs. site read: 0.1315; CT-FFR vs. AI-QCT: 0.0884; AI-QCT vs. site read: 0.0004327. AUC, area under the curve; CT-FFR, coronary computed tomography angiography using fractional flow reserve; AI-QCT, artificial intelligence coronary computed tomography.

AI-QCT_ISCHEMIA_ demonstrated a specificity of 0.74 (95% CI 0.57–0.88) vs. 0.51 (95% CI 0.34–0.69) for CT-FFR and 0.17 (95% CI 0.07–0.36) for physician visual interpretation. AI-QCT_ISCHEMIA_ revealed a sensitivity of 0.84 (0.65–0.97) vs. 0.84 (95% CI 0.60–0.97) for CT-FFR and 1.00 (95% CI 1.00) for physician visual interpretation. AI-QCT_ISCHEMIA_ demonstrated a PPV of 0.64 (95% CI 0.54–0.79) vs. 0.48 (95% CI 0.39–0.58) for CT-FFR and 0.40 (95% CI 0.36–0.43) for physician visual interpretation. AI-QCT_ISCHEMIA_ demonstrated a NPV of 0.90 (95% CI 0.75–0.96) vs. 0.86 (95% CI 0.70–0.95) for CT-FFR and 1.00 (95% CI 1.00) for physician visual interpretation. The diagnostic accuracy was 0.78 (95% CI 0.66–0.89) for AI-QCT_ISCHEMIA_ vs. 0.63 (95% CI 0.49–0.76) for CT-FFR and 0.46 (95% CI 0.33–0.60) for physician visual interpretation. The area under the curve (AUC) for AI-QCT_ISCHEMIA_ was 0.91 vs. 0.76 (*P* value = 0.09) for CT-FFR and 0.62 (*P* value < 0.01) for physician visual interpretation as shown in *Figure 3*.

The McNemar test performed for CT-FFR vs. ICA-FFR showed a *P* value of 0.004, for visual interpretation CAD-RADS vs. ICA-FFR showed a *P* value of 1.99 × 10^−7^, and for AI-QCT_ISCHEMIA_ vs. ICA-FFR showed a *P* value of 0.146. The NIR performed for CT-FFR vs. ICA-FFR showed a *P* value (accuracy > NIR) = 0.67, for visual interpretation CAD-RADS vs. ICA-FFR showed a *P* value (accuracy > NIR) = 0.998, and for AI-QCT_ISCHEMIA_ vs. ICA-FFR showed a *P* value (accuracy > NIR) = 0.029.

When comparing the accuracy performance of CT-FFR at various thresholds, the analysis of CT-FFR ≤ 0.8 revealed 16 true positive vessels out of a total of 33 positive vessels, with a PPV of 0.48. CT-FFR ≤ 0.7 revealed 10 true positives out of a total of 17 positives with a PPV of 0.59. For CT-FFR between 0.7 and 0.8, there were 6 true positives out of a total of 16 positives with a PPV of 0.375.

When comparing expert physician visual interpretation based on CAD-RADS ≥ 3 to the gold standard adenosine ICA-FFR, out of a total of 48 physician-read positive vessels, there were 19 vessels that were true positives and 29 that were false positives. Out of a total of 6 negative vessels, there were 0 false negative vessels and 6 true negatives. Given that there were no false negatives for physician visual interpretation, the sensitivity and NPV were both 1.0 (*Table 2*).

### LAD sub-analysis

The sub-analysis focusing on the LAD showed there were 15 true positives, 11 false positives, 0 false negatives, and 3 true negatives for physician visual interpretation CAD-RADS; 12 true positives, 8 false positive, 3 false negatives, and 6 true negatives for CT-FFR; and 14 true positives, 3 false positives, 1 false negative, and 11 true negatives for AI-QCT.

Based on this LAD sub-analysis, in comparison to CT-FFR and visual interpretation by expert physicians, AI-QCT exhibited higher values in all statistical measures including diagnostic accuracy, sensitivity, specificity, PPV, NPV, and AUC. These values can be found in *Table 3*.

**Table 3:** Comparing per-vessel imaging modalities vs. adenosine ICA-FFR for LAD

Metric (95% confidence interval)	Physician visual interpretation CAD-RADS ≥ 3	CT-FFR	AI-QCT_ISCHEMIA_
Diagnostic accuracy	0.62	0.62	0.86
Sensitivity TP/(TP + FN)	1.00 15/15	0.80 12/15	0.93 14/15
Specificity TN/(FP + TN)	0.21 3/14	0.43 6/14	0.79 11/14
Positive predictive value (PPV) TP/(TP + FP)	0.58 15/26	0.60 12/20	0.82 14/17
Negative predictive value (NPV) TN/(TN + FN)	1.00 3/3	0.67 6/9	0.92 11/12
Area under the curve (AUC)	0.62	0.62	0.86

TP, true positive; TN, true negative; FP, false positive; FN, false negative.

### Plaque characteristics analysis

The plaque characteristics that were different in false positive vs. true positive cases for CT-FFR were maximum % stenosis (40% vs. 65%, *P* < 0.001) and total non-calcified plaque (9% vs. 13%, *P* < 0.01); for physician visual interpretation, they were total non-calcified plaque (8% vs. 12%, *P* < 0.01), lumen volume (681 vs. 510 mm^3^, *P* = 0.02), maximum % stenosis (40% vs. 62%, *P* < 0.001), total plaque (19% vs. 33%, *P* = 0.002), and total calcified plaque (11% vs. 22%, *P* = 0.003); and for AI-QCT_ISCHEMIA_ was maximum % stenosis (54% vs. 67%, *P* < 0.01). Additional comparison plaque characteristics of false positives vs. true positive among these modalities can be found in *Table 4*.

**Table 4:** Comparing plaque characteristics of false positives vs. true positive for CT-FFR, physician visual interpretation CAD-RADS, and AI-QCT_ISCHEMIA_

Physician visual interpretation CAD-RADS				
Plaque characteristic (mean)	False positive (*n* = 29)	True positive (*n* = 19)	Overall (*n* = 48)	*P* value
Low density plaque (%)	0.021	0.013	0.018	0.6980
Lumen volume	681.176	510.758	613.719	0.0228
Maximum stenosis diameter percentage	0.407	0.619	0.491	0.0000
Total calcified plaque (%)	10.666	21.517	14.961	0.0031
Total non-calcified plaque (%)	8.422	11.849	9.779	0.0072
Total plaque (%)	19.088	33.366	24.74	0.0016
CT-FFR				
Plaque characteristic (mean)	False positive (*n* = 17)	True positive (*n* = 16)	Overall (*n* = 33)	*P* value
Low density plaque (%)	0.026	0.015	0.021	0.774
Lumen volume	636.682	512.212	576.333	0.0562
Maximum stenosis diameter percentage	0.401	0.654	0.524	0.0001
Total calcified plaque (%)	14.217	21.563	17.779	0.1210
Total non-calcified plaque (%)	8.965	12.954	10.899	0.0095
Total plaque (%)	23.182	34.517	28.678	0.0367
AI-QCT				
Plaque characteristic (mean)	False positive (*n* = 9)	True positive (*n* = 16)	Overall (*n* = 25)	*P* value
Low density plaque (%)	0.008	0.015	0.012	0.5850
Lumen volume	657.633	550.094	588.808	0.1070
Maximum stenosis diameter percentage	0.541	0.672	0.625	0.0042
Total calcified plaque (%)	18.777	20.659	19.981	0.6100
Total non-calcified plaque (%)	13.553	12.168	12.666	0.6510
Total plaque (%)	32.33	32.827	32.648	0.9100

## Discussion

**Figure 4:**
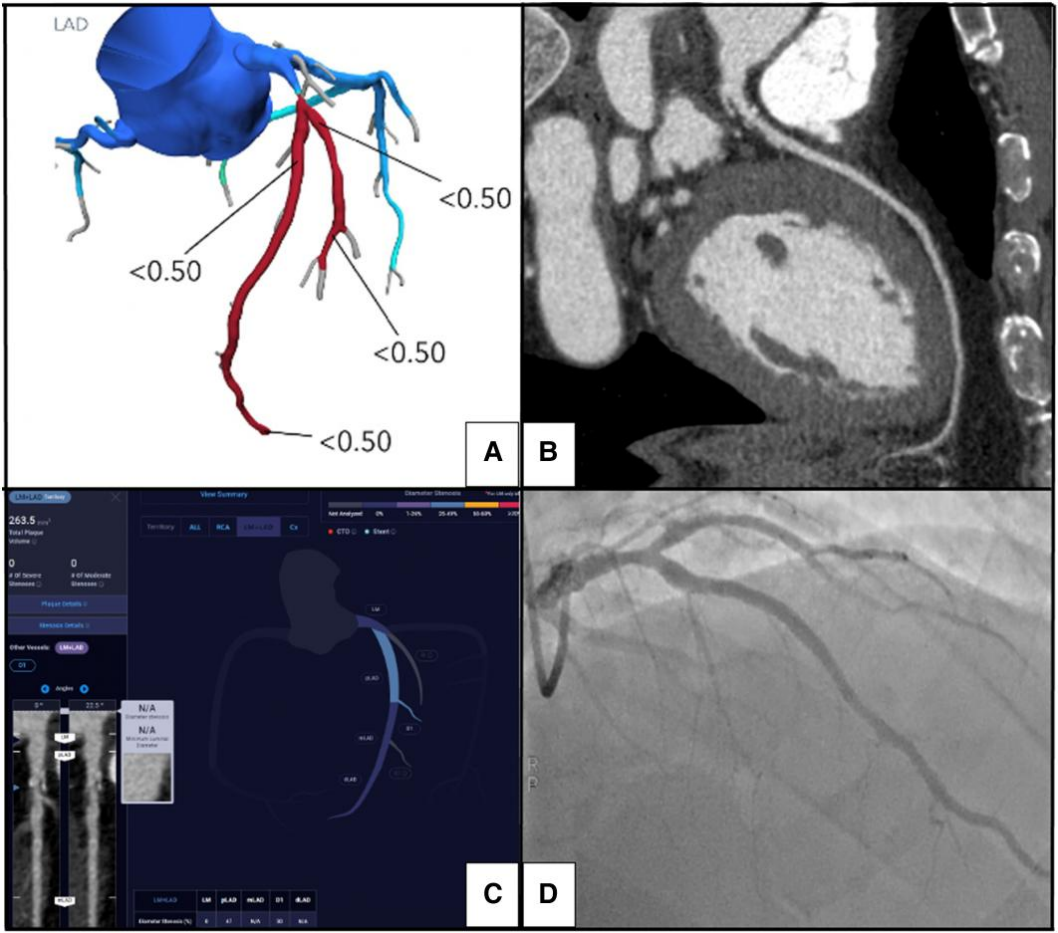
Representative example of all metrics assessed including CT-FFR, expert physician visual interpretation of CAD-RADS, AI-QCT_ISCHEMIA_, and ICA. (*A*) CT-FFR analysis of proximal LAD with a value < 0.50; (*B*) expert physician interpretation of CCTA showing moderate stenosis proximal LAD with MD read of CAD-RADS 3; (*C*) AI-QCT analysis of proximal LAD reading mild stenosis without significant ischaemia; (*D*) ICA of the proximal LAD with mild disease and physiologically non-significant ICA-FFR value of 0.95. CT-FFR, coronary computed tomography angiography using fractional flow reserve; AI-QCT, artificial intelligence coronary computed tomography; ICA, invasive coronary angiography.

Our study is one of the first to investigate the diagnostic accuracy of AI-QCT_ISCHEMIA_ as a first line detection tool for myocardial ischaemia by comparing the performance of AI-QCT_ISCHEMIA_, CT-FFR, and physician visual interpretation CAD-RADS against gold standard adenosine ICA-FFR (*Figure 4*). AI derived coronary plaque characteristics were analysed for each technique.

### Diagnostic performance

Each of the three techniques was individually compared with the gold standard ICA-FFR. On the outcome of per-vessel prediction of FFR ≤ 0.80, AI-QCT_ISCHEMIA_ had a higher specificity, PPV, accuracy, and AUC vs. CT-FFR and physician visual interpretation CAD-RADS ≥ 3 (*Table 2*, *Figure 3*). Based on the McNemar test and NIR, AI-QCT_ISCHEMIA_ was shown to be most significantly similar to ICA-FFR regarding diagnostic performance.

The diagnostic performance of AI-QCT_ISCHEMIA_ was developed and validated within the CREDENCE and PACIFIC study cohorts that included patients undergoing CCTA and used FFR of all coronary arteries as gold standard. In 305 patients and 868 vessels of CREDENCE, the AUC for AI-QCT_ISCHEMIA_ on a per-vessel basis was 0.86 (0.84–0.89) compared with 0.76 (0.72–0.80) for CT-FFR (*P* < 0.001).^17^ Further, as external validation in PACIFIC (208 patients, 612 vessels), the AUC for AI-QCT_ISCHEMIA_ was 0.86 (0.83–0.89) compared with 0.83 (0.79–0.87) for CT-FFR (*P* = 0.062). The per-vessel calcified plaque volume in CREDENCE was higher than PACIFIC (middle tertile range 11–64.7 mm^3^ vs. 0.5–14.7 mm^3^).^18^

The trend towards improved CT-FFR per-vessel PPV with lower thresholds for optimal cut-off value was also found by Matsumura-Nakano *et al*.^19^ in a study of 139 vessels. The per-vessel accuracy of CT-FFR for invasive FFR ≤ 0.8 was 32% in the cases with range of FFR_CT_ = 0.71–0.80. They proposed a grey zone of FFR_CT_ of 0.71–0.80, with resultant high PPV (82%) in the range of FFR_CT_ ≤ 0.70 and high NPV (94%) in the range of FFR_CT_ > 0.80. Sixteen out of 33 of our positive CT-FFR vessels were between 0.70 and 0.80, therefore nearly half of all our positive vessels would be rendered indeterminate of ischaemia.

### Comparison of rate of false positives and true positives for each technique

False positive results for expert physician visual interpretation, CT-FFR, and AI-QCT_ISCHEMIA_ when each was compared with ICA-FFR were 29, 17, and 9 false positives, respectively. Notably, this all-comers study of patients referred to the cath lab after a positive CCTA and positive CT-FFR had a high median total CAC score of 654. Although CT-FFR analysis has been shown to be clinically useful in patients across a wide range of CAC severity, as expected, markedly elevated levels of intra-vessel CAC lowers per-vessel accuracy (*Figure 1*). Factors that may have contributed to these false positive cases with AI-QCT_ISCHEMIA_ include a high CAC inherent to this study resulting in calcium blooming CCTA partial volume artefacts. Although the AUC of AI-QCT_ISCHEMIA_ is lower with increasing tertiles of calcified plaque in a CREDENCE substudy, it remained substantially higher than CT-FFR and MPI.^18^

Finally, we compared the plaque characteristics of false positive vs. true positive results for physician visual interpretation CAD-RADS, CT-FFR, and AI-QCT_ISCHEMIA_, we found that the greater the plaque and stenosis, the more likely a true positive result with CT-FFR and physician visual interpretation; and for AI-QCT_ISCHEMIA_, greater stenosis.

### Limitations

The small sample size of this single-centre study of 44 patients and 54 vessels results in limited statistical power and wide 95% confidence limits. Despite this limitation, the overall conclusions are concordant with CREDENCE-PACIFIC substudy.^16,18,20^

Post-test referral selection bias and verification biases are a limitation of this observational diagnostic study. Of the 3291 CCTA patients referred for CCTA in this time period, CT-FFR analysis was chosen by two conditions: physician visual interpretation CAD-RADS ≥ 3, AND the availability of reimbursement for the CT-FFR procedure. No patients with FFR-CT > 0.80 were evaluated by ICA. The decision to perform invasive FFR was based on physician preferences. Since cases chosen for invasive FFR were based on a positive CT-FFR, this study cannot provide a good estimate of the true negative predictive value. Furthermore, for the final analysis, only those positive CT-FFR patients referred for ICA who had adenosine FFR performed in at least one vessel were included. Use of non-adenosine ICA-FFR techniques was not included in this study. In the publication by Mittal *et al*.^21^, where the NHS provided CT-FFR without requirement for an ability-to-pay, the per-patient PPV for CT-FFR was found in 213 patients as 49%. The selection criteria in this NHS study as well as our study reflect the real-world realities in clinical practice.

Another aspect of selection bias regards the PPV of AI-QCT_ISCHEMIA_. Our population was selected by physician read ≥ CAD-RADS 3 stenosis and positive CT-FFR and ICA adenosine FFR measurement. The true PPV of AI-QCT_ISCHEMIA_ was not determined, since all 442 patients in the FFR_CT_ population did not undergo AI-QCT. For instance, false positive values by AI-QCT that were negative by CT-FFR would be missed. The true PPV for AI-QCT without such bias is found in the CREDENCE-PACIFIC substudy.^16,18,20^

The reported performance of all three tests is based upon a sample population restricted to those patients with physician interpreted CAD-RADS 3 or greater CAD. This is a skewed population and cannot possibly represent a normal distribution of all positive and negative results for FFR less than or >0.80 in the derivative population of all 3191 coronary CT angiograms. Nonetheless, this reflects the real world usage of advanced quantitative CT analysis in CAD.

This was a real-world study, and so-called core laboratory ‘co-location’ analysis of CT-FFR correlated with ICA and FFR was not performed. We used the vendor’s reporting of CT-FFR value 10 mm distal to the stenosis.^13^ The study results are hypothesis generating and requires further prospective evaluation with a larger cohort of patients.

## Conclusion

When AI-QCT_ISCHEMIA_, CT-FFR, and visual interpretation by expert physicians were individually compared with the gold standard of adenosine ICA-FFR, AI-QCT_ISCHEMIA_ exhibited higher specificity, PPV, accuracy, and AUC and had a diagnostic performance most statistically similar to ICA-FFR. Plaque characteristic analysis revealed that the greater the plaque and stenosis, the more likely a true positive result with CT-FFR and physician visual interpretation; and for AI-QCT_ISCHEMIA_, greater stenosis.

## Data Availability

The data underlying this article are available in the article.

## Lead author biography

Dr Andrew Chiou is a cardiology fellow currently at Scripps Clinic in San Diego, CA. Dr Chiou earned his Bachelor of Science degree at Duke University and his academic interests paved the way for him to pursue his Doctor of Medicine degree at the University of Nebraska Medical Center. His post-graduate training commenced with an internal medicine residency at the University of California, Davis Medical Center prior to entering cardiology fellowship. Dr Chiou is committed towards research focusing on the diagnosis and evaluation of ischaemic disease. He is interested in a career in Interventional and Structural Cardiology.

## Appendix

A Pearson correlation analysis conducted for CT-FFR against ICA-FFR revealed a Pearson correlation coefficient (*R*) of 0.369 and *P* value of 0.006. The Bland–Altman analysis showed a mean difference of 0.06 (95% CI −0.19–0.32) between CT-FFR and ICA-FFR.
